# Age, period and cohort effects in depression prevalence among Canadians 65+, 1994 to 2018: A multi-level analysis

**DOI:** 10.1177/00207640221141785

**Published:** 2022-12-07

**Authors:** Guang Yang, Carl D’Arcy

**Affiliations:** 1 School of Public Health, University of Saskatchewan, Saskatoon, Saskatchewan, Canada; 2 Department of Psychiatry, College of Medicine, University of Saskatchewan, Saskatoon, Saskatchewan, Canada

**Keywords:** age-period-cohort effect, major depressive episode, hierarchical linear models, older adults

## Abstract

**Background:**

The nature of the Canadian population 65+ has changed considerably over the past several decades. They comprise a larger proportion of the population, are better educated, and are wealthier than previous generations. We estimate the contributions of chronological aging, temporal periods, and birth cohort effects on the trends in the major depressive episode (MDE) prevalence among Canadian seniors from 1994/1995 to 2017/2018.

**Methods:**

Using data from two sets of national health surveys, the National Population Health Survey (NPHS) and the Canadian Community Health Survey (CCHS). Pooled data on 150,246 survey respondents aged 65+ from 16 repeated cross-sectional surveys are included. Hierarchical regression age-period-cohort models were used to visualize the linear and non-linear effects of age, period, and cohort trends in late-life depression.

**Results:**

We found that: the prevalence of MDE in later life fluctuated non-significantly during the study time period; the probability of developing MDE declined with increasing age from 65 to 80+ (β = −.32, *p* = .027). The significant quadratic birth cohort predictor showed a non-linear increasing association with the prevalence of MDE from the earlier to later-born cohorts (β = .01, *p* = .049). We also found that females 65+ were consistently more likely to be depressed than males 65+ (β = .47, *p* = .007). The significantly negative “age × female” interaction shows that age exerts a greater effect on females’ probability of developing MDE than males (β = −.09, *p* = .011). There were no consistent significant period effects but there were peaks in prevalence around 2001, 2008, and 2012 which corresponded to some recent historical events. Our moderation analysis documents that lower levels of education significantly contributed to the higher rates of depression among cohorts born earlier in the 20th century.

**Conclusions:**

Our findings show the presence of strong chronological age and cohort effects and weaker period effects on the prevalence of late-life depression in Canadian seniors.

## Introduction

Rapid growth in the number and proportion of older adults globally makes attention to mental health and aging timely and significant. Depression is a major public health problem throughout the lifespan, particularly in older age. The prevalence of depression in older people has been reported to vary broadly, from 8.2% to 63.0% globally (Hu et al., 2022), due to differences in research areas, screening instruments, and levels of public health services (Krishnamoorthy et al., 2020; Moreno-Agostino et al., 2021). Depression in later life leads to significant distress and has been related to several adverse outcomes, such as functional impairment and increased suicide rates. In addition, late-life depression has been linked to an increased likelihood of death from chronic diseases, including dementia (Li et al., 2019).

Depression is a treatable health condition. It is not a typical manifestation of aging. However, a fair share of studies show that depression symptoms tend to increase as we age (Aziz & Steffens, 2013; Bell, 2014). Late-life depressive symptoms are frequently confused with the effects of multiple chronic diseases and the medicines used to treat them. Because older people often exhibit depressive symptoms differently from younger adults, doctors and families may miss these symptoms. For example, when older adults begin to display specific symptoms, such as insomnia, weight loss, gastrointestinal distress, and fatigue, they are often perceived as part of normal aging (Wu et al., 2012). Furthermore, advancing age is often accompanied by the loss of social support due to the death of a life partner or siblings and friends, retirement, or relocation. Late-life mental health problems remind us that a better understanding of how depression and risk factors change among old-age adults is of value for clinical and public health decision-making, especially in the 21st century, when individuals are increasingly living longer.

It is well-known that inequalities in mental health have social determinants. Mental health inequalities not only result from specific characteristics and events but are closely related to structural factors, such as living arrangements, social-economic status, social and health policies, and legislation, and other dynamic facets that influence mental health outcomes in societies (Fryers et al., 2003). Variations in the prevalence of late-life depression over time can be elucidated in light of 3 time-related effects: age, temporal period, or birth cohort. First, changes in depression prevalence can be seen as a result of the aging process. Zhao et al. (2012), in a meta-analysis, reported that the prevalence of depression generally increases with age among the elderly. Extensive research has illustrated that mental health is dynamic, and risk factors accumulate throughout the life course, with factors in childhood adding to contemporary factors to affect depression later in life (Su et al., 2022; Wirback et al., 2014). Depression is linked to biological and social processes related to aging. Second, variations can be due to a range of environmental, social, or economic factors affecting older adults of all ages during a particular period, such as war, famine, financial crises, or new treatment methods. Third, depression prevalence could be the result of cohort effects, conceptualized as resulting from the unique experiences or exposures of a cohort as they move through time—typically a birth cohort in which the disease arises from an exposure unique to that cohort (Keyes et al., 2010).

In this paper, we seek to explore the prevalence of depression among Canadians 65+ during the years 1994 to 2018. We use an age-period-cohort (APC) approach to investigate to what extent trends in the prevalence of late-life depression over time are due to period or birth cohort and chronological age effects.

## Methods

### Study design and data source

This study utilizes data from the National Population Health Survey (NPHS) (Statistics Canada, 2013), and the general health cycles of the Canadian Community Health Survey (CCHS) (Statistics Canada, 2019). The NPHS was a longitudinal study beginning in 1994 and conducted every 2 years (in 1994/1995, 1996/1997, and 1998/1999). The NPHS contains both nationally representative cross-sectional and longitudinal samples. The CCHS omnibus health survey, a successor to the NPHS, is an ongoing series of cross-sectional general health surveys starting in 2001. Since Canada is a confederation of provinces and territories and health is constitutionally a provincial responsibility, all items asked in the annual health surveys are not necessarily asked in all provinces or territories. Consequently, the CCHS contains both core contents asked in all provinces and territories and optional content modules that provinces and territories can opt into or out of in any collection year. The epidemiological measurement of depression was optional in the CCHS general surveys. So, provinces or territories can opt in or out of collecting data on depression; thus, sample sizes, while considerable, are variable in any given survey year. Supplemental Appendix Table 1 provides details on the data collected on depression in each survey/year and the survey sample size for respondents 65+ sample. The total number of survey respondents 65+ over the study time period is 362,154. Survey respondents for whom depression data were missing were excluded from the analysis. For partially missing data, we performed five rounds of imputation based on a fully conditional specification model (Markov chain Monte Carlo) (Takahashi, 2017). The multiple imputations were implemented using the R MICE (Multivariate Imputation via Chained Equations) package. The final sample studied was 150,246 survey participants.

### Measures

Major depressive episode (MDE) in the surveys was assessed by two different but well-established instruments frequently employed in epidemiological surveys. The World Health Organization Composite Diagnostic Inventory Short Form for major depressive episode (CIDI-SFMD) was used to assess depression in the last 12 months in NPHS surveys of 1994/1995, 1996/1997, and 1998/1999, and in the CCHS surveys of 2001, 2003, 2005, 2007/2008, 2008/2009, 2009/2010, 2010, 2011/2012, 2012, 2013/2014, and 2014. For the CIDI-SFMD, a score of 5 or more is used as evidence of a major depressive episode (MDE) in the past year; they were coded as 1, and others as 0 (Kessler et al., 1998). The Patient Health Questionnaire (PHQ-9), a quick depression assessment tool, was used in CCHS surveys of 2015/2016 and 2017/2018 to assess MDE in the past 2 weeks. A cut-off score of ⩾10 on the PHQ-9 was used as the criterion for the presence of MDE (He et al., 2020).

*Periods* were defined by the year in which the survey was conducted between 1994 and 2018.

The measurement of *chronological age* is years since birth. Survey participants (all 65+) were categorized into four age groups: 65 to 69, 70 to 74, 75 to 79, and 80+.

*Birth cohorts* were calculated as survey year (period) minus the respondents’ age. They were categorized as follows: 1910 to 1914, 1915 to 1919, 1920 to 1924, 1925 to 1929, 1930 to 1934, 1935 to 1939, 1940 to 1949, and 1950 to 1954.

In the current study we controlled for *gender* (male/female), *province of residence* (British Columbia/Alberta/Saskatchewan/Manitoba/Ontario/Quebec/New Brunswick/Nova Scotia/Prince Edward Island/Newfoundland and Labrador/Yukon Territory/Northwest Territories/Nunavut), *household income* (lowest/lower/middle/upper/highest income quintile), and *educational attainment* (less than secondary/secondary graduation/some post-secondary/post-secondary graduation).

### Statistical analysis

We first conducted descriptive analyses to summarize the age-specific prevalence of MDE among Canadians 65+ from 1994/1995 to 2017/2018. Next, to better separate the effects of age, temporal period, and birth cohort, we performed APC analyses. However, the identification problem is a crucial methodological challenge when considering temporal aspects of the study variables (Bell & Jones, 2015). It arises because the three variables of age, period, and birth cohort are collinear, and it is difficult to separate out their distinct effects. Thus, we performed a Hierarchical APC (HAPC) model, a cross-classified multilevel model that can be used for repeated-cross-sectional data (Yang & Land, 2013). Although some simulation studies show that the HAPC approach cannot circumvent the identification problem perfectly (Bell & Jones, 2014), this model presents a compelling conceptualization of APC (Bell, 2014). Given the extensive debate about the critique of the HAPC model, we interpret our results with caution. Bell and Jones’ study supports the view that the range of the periods and cohorts set by the data structure drives the accuracy of results (Bell & Jones, 2018). Therefore, our modeling strategy favors cohort over period trends in order to deal with the identification problem. This multilevel model is intuitive, and we extended this HAPC model to incorporate other fixed parts in the current analyses. We included quadratic age and cohort terms to account for the curvilinear relationship. Following the recommendations of Yang and Land (Yang & Land, 2013), we modeled the categorical dependent variable using three logistic regression models with random intercepts for periods and cohorts. We extended these models by including a cohort polynomial in the fixed part. Thus, fixed age and birth cohort effects describe the overall variation in the outcome attributable to age and birth cohort, whereas random period and cohort effects describe the overall variations in the intercept of the model across periods and cohorts.

Model 1 adjusts for gender and province of residence; Model 2 adjusts for gender, province of residence, and household income; Model 3 adjusts for gender, province of residence, household income, and educational attainment.

All HAPC analyses were conducted using the “lme4” package in R (Stegmann et al., 2018). Sampling weighting cannot be used for random effects analyses because pairwise selection probabilities are required when estimating proper probability weighting for variance components, which is inappropriate using current statistical software (Twenge et al., 2019). The Akaike information criterion (AIC) and Bayesian information criterion (BIC) statistics are used to evaluate the goodness of fit (Singer et al., 2003). Graphs were generated in OriginPro 8.0 software (OriginLab Corporation, Northampton, MA, USA) (Moberly et al., 2018).

## Results

### Descriptive analyses

The trends in the prevalence of MDE for all age groups 65+ are shown in Supplemental Appendix Figure 1A. From 1994 to 2018, the prevalence of MDE fluctuated non-significantly over time. Several peaks are evident around the years 2001 (3.32%), 2008 (3.22%), and 2011 to 2012 (3.85%), especially for the 65 to 69 and the 70 to 74 age groups (see Supplemental Appendix Figure 1B). Prevalence of MDE was highest among 65 to 69 age group and among women in comparison to men older adults.

Supplemental Appendix Figure 2 shows the age-specific trend by period and cohort-specific prevalence by age group. For most of the periods, the prevalence of depression decreased gradually with age. Supplemental Appendix Figure 2B shows that age groups do not share the same pattern in different birth cohort groups. For people aged 70 +, the prevalence of MDE was higher in those born earlier in the 20th century than in those born later, whereas the opposite was true for the 65 to 69 age group as the prevalence of MDE in this age group increases in more recent birth cohorts. The “baby boom” generation born after the end of World War II are part of the 1945 to 1949 and 1950 to 1954 birth cohorts.

### Hierarchical APC results

**Figure 1.**
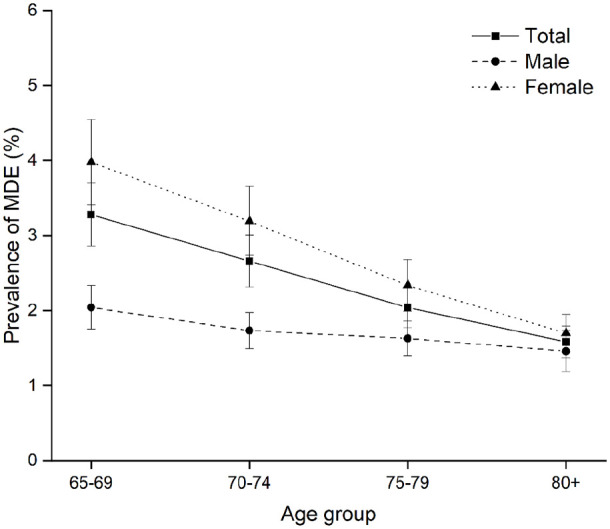
Predicted prevalence of MDE among Canadians 65+, males and females, by age group, 1994 to 2018.

**Figure 2.**
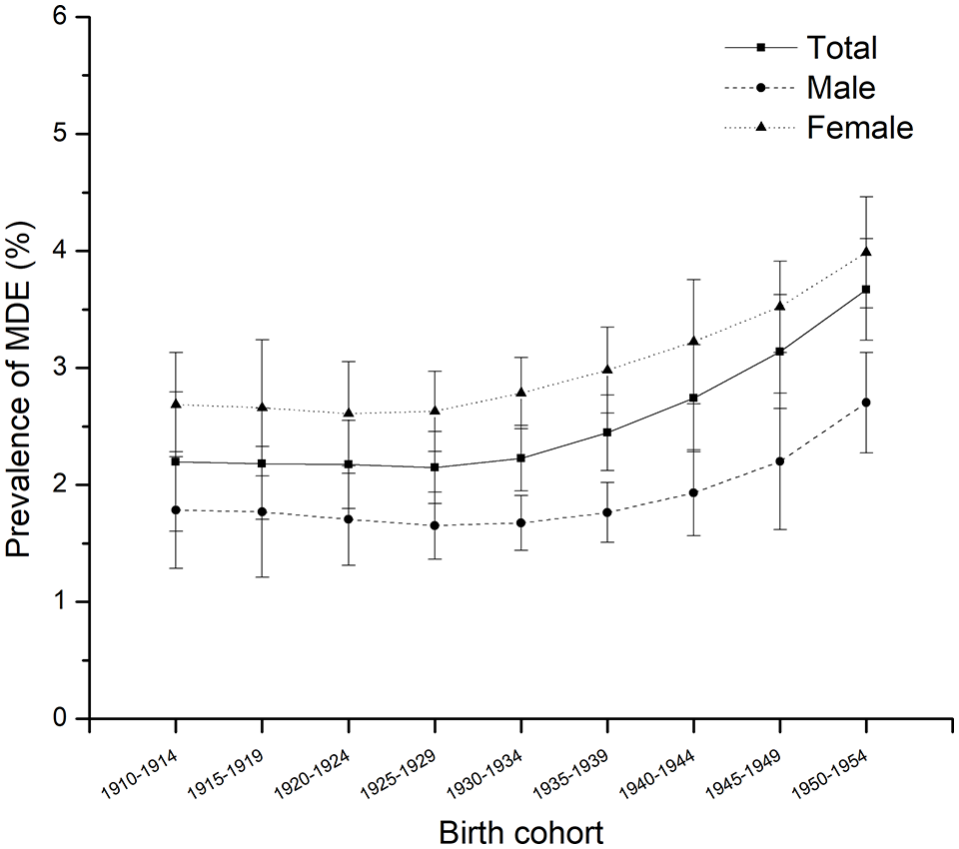
Predicted prevalence of MDE among Canadians 65+, males and females by birth cohort.

**Figure 3.**
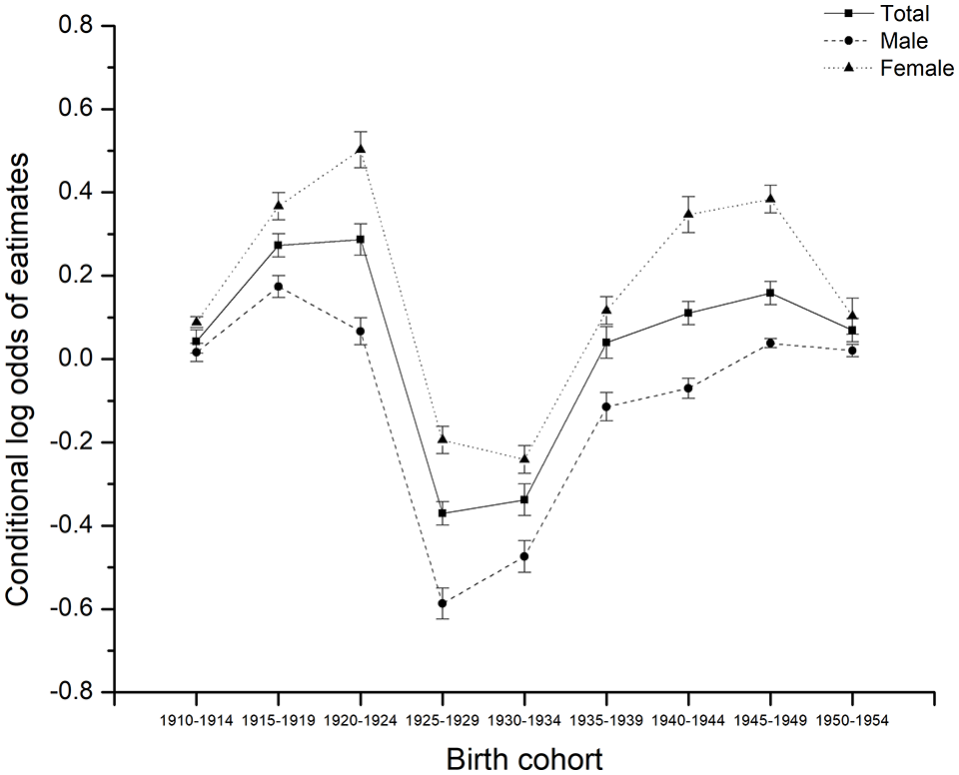
Parameters estimated for cohort effects by age-period-cohort modeling for the prevalence of MDE among Canadians 65+ by sex, 1994 to 2018.

**Figure 4.**
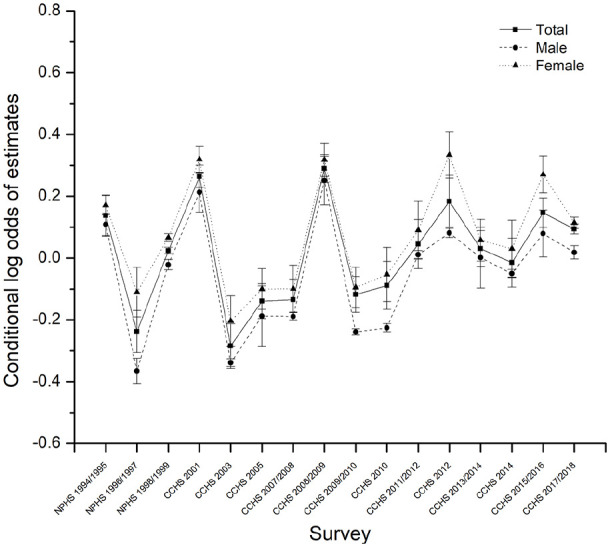
Parameters estimated for period effects by age-period-cohort modeling for the prevalence of MDE among Canadians 65+s by sex, 1994 to 2018.

We conducted a hierarchical regression analysis to decompose linear chronological age and birth cohort trends and random period and birth cohort fluctuations in the APC analysis. We also entered some focal variables stepwise to test the moderation effects of household income and educational attainment in these models. The parameters estimated by APC models are illustrated in Table 1. The gender difference in the age-MDE association is accounted for by adding an “age × sex” interaction term in these models. Figures 1 and 2 show the fixed effects of age and cohort on the likelihood of developing MDE. The deviations of periods and cohorts from predictions of the fixed part in model 3 were displayed in Figures 3 and 4. The birth cohort random intercepts represent the unexplained variation left after controlling for the cohort trend and individual-level predictors. All random intercept variances are significant on a *p* < .05 level.

**Table 1.** Age, period, and cohort effects of MDE among Canadians 65+.

	Model 1			Model 2			Model 3		
	β	*SE*	*p*	β	*SE*	*p*	β	*SE*	*p*
Fixed effects									
Intercept	−3.58	0.16	<.001	−3.80	0.16	<.001	−3.70	0.16	<.001
Age	−.32	0.15	.027*	−.28	0.15	.049*	−0.29	0.15	.043*
Age (squared)	.05	0.03	.087	.03	0.03	.289	.03	0.03	.281
Gender (female)	.47	0.18	.007**	.40	0.18	.026*	.41	0.18	.023*
Age × female	−.09	0.03	.011*	−.07	0.04	.032*	−0.07	0.03	.03*
Age^2^ × female	.27	0.17	.120	.17	0.17	.319	.17	0.17	.325
Cohort	−.02	0.07	.691	.02	0.35	.329	.14	0.34	.258
Cohort (squared)	.01	0.02	.049*	.01	0.04	.043*	−0.20	0.23	.039*
Household income: lowest income quintile									
Lower income quintile				−.34	0.04	<.001	−0.32	0.04	<.001
Middle income quintile				−.57	0.05	<.001	−0.54	0.05	<.001
Upper income quintile				−.61	0.07	<.001	−0.58	0.06	<.001
Highest income quintile				−.76	0.07	<.001	−0.74	0.07	<.001
Education: Less than secondary school graduation									
Secondary school graduation, no post-secondary education							−0.23	0.05	<.001
Post-secondary certificate diploma or univ degree							−0.11	0.04	.002**
Province: Newfoundland									
Prince Edward Island	−.01	0.12	.940	−.01	0.12	.960	.02	0.12	.882
Nova scotia	.41	0.10	<.001	.40	0.10	<.001	.42	0.10	<.001
New Brunswick	.23	0.11	.035*	.23	0.11	.036*	.24	0.11	.022*
Quebec	.06	0.09	.500	.04	0.09	.650	.05	0.09	.596
Ontario	.09	0.09	.289	.10	0.11	.289	.14	0.09	.123
Manitoba	.09	0.11	.351	−.11	0.10	.342	.12	0.11	.257
Saskatchewan	−.10	0.11	.330	.12	0.10	.311	−.08	0.10	.432
Alberta	.12	0.10	.231	.54	0.09	.217	.16	0.08	.109
British Columbia	.54	0.09	<.001	.12	0.29	<.001	.60	0.28	<.001
Yukon/Northwest Territories/Nunavut	.18	0.28	.522	.18	0.22	.342	.38	0.26	.456
	Variance	Std.Dev		Variance	Std.Dev		Variance	Std.Dev	
Random effects									
Period	.048	0.220		.052	0.228		.053	0.231	
Cohort	.001	0.001		.001	0.001		.001	0.001	
Summary statistics									
AIC	35,786.4			35,427.1			35,406.9		
BIC	35,865.7			35,526.3			35,525.9		

*Note*. Model 1 is adjusted for gender only (fixed effects); Model 2 is adjusted for age, gender, marital status, educational status, and BMI.

*SE* = standard error.

* *p* < .05. **p < .005

We investigated fixed age and cohort effects as well as random period and cohort effects controlling for gender and province of residence in model 1. Predictions for age are in accordance with the graphic impression from the descriptive analysis. The fixed effect coefficient for age is negative and significant, indicating that the probability of developing MDE declined from 65 to 80+ (β = −.32, *p* = .027). This could be interpreted as evidence of a healthy survivor effect. As expected, females 65+ were more likely to be depressed than males 65+ (β = .47, *p* = .007). Figure 3 graphically displays the predicted prevalence of MDE depending on age and sex. While there is a similar decreasing trend in the age effect for both males and females, it is more pronounced among females. The significantly negative “age × female” interaction predicts that age exerts a greater effect on females’ probability of developing MDE than males (β = −.09, *p* = .011).

Controlling for the age effect, the significantly quadratic cohort predictor shows a nonlinear increasing association with the prevalence of MDE from the earlier to later-born cohorts (β = .01, *p* = .049). Figure 2 visualizes the fixed cohort trends of the probability of developing MDE. We see that the earliest cohorts report a much probability of MDE (close to 2.6% in the 1910–1914 birth cohort), which is lowered slightly in later cohorts (down to 2.07% in the 1925–1929 birth cohort), and then substantially rises again in recent birth cohorts, to 3.67% in the 1950 to 1954 birth cohort. Figure 3 graphically displays the random cohort effects, which fluctuated non-significantly. As mentioned earlier, these random intercepts correspond to the unexplained variation after accounting for the cohort trend. The random cohort effects trend downwards from 1920 to 1924 to 1925 to 1929 birth cohorts and then move upward in the 1940 to 1944 and later birth cohorts.

The period random effects are displayed in Figure 4. The overall period random intercept fluctuated non-significantly except for some peaks around 2001, 2008, and 2012. This is consistent with the descriptive results (Supplemental Appendix Figure 1A).

**Figure 5.**
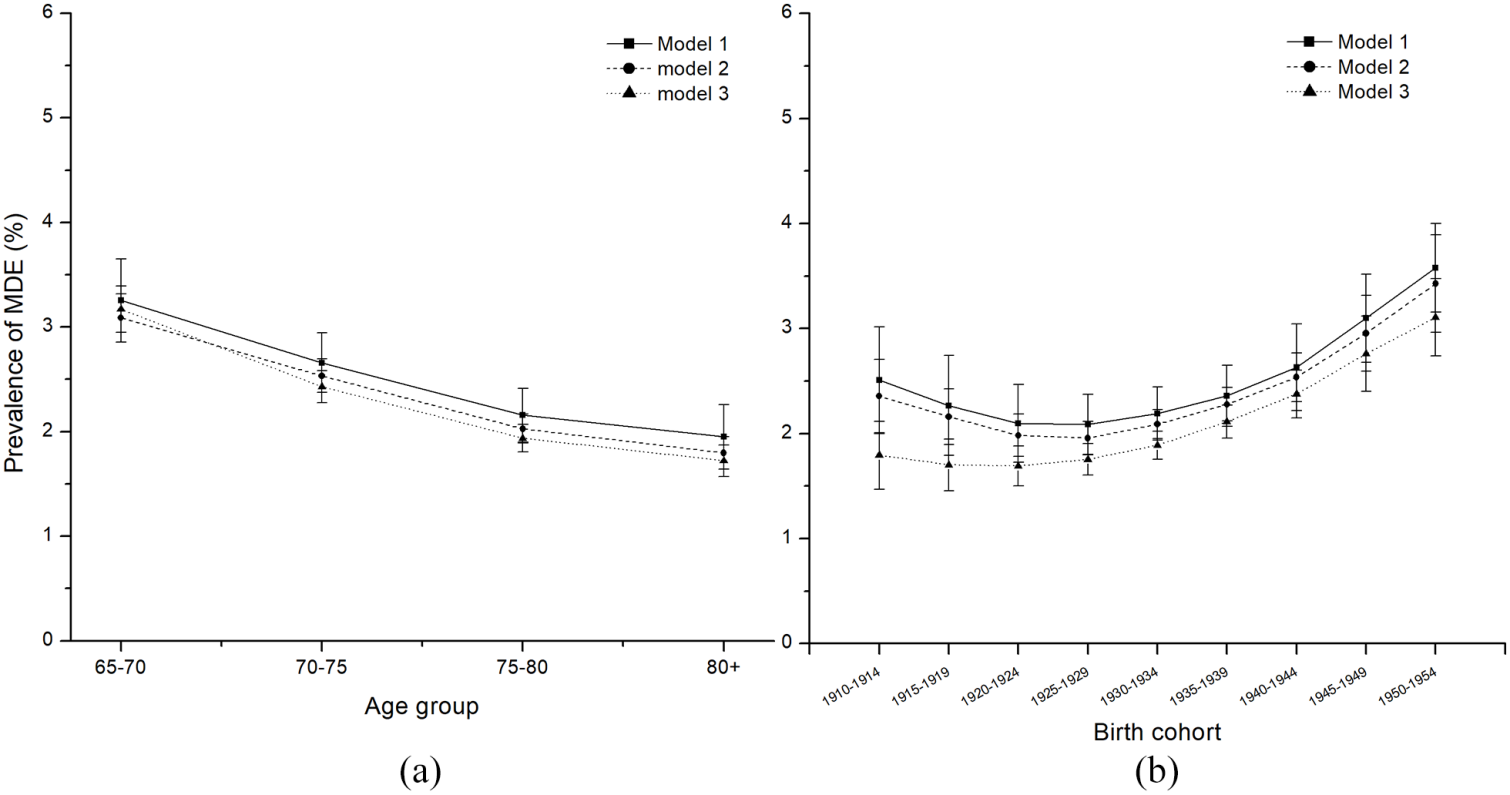
Predicted prevalence of MDE among Canadians 65+. based on models 1–3 for age (a) and birth cohort (b), 1994 to 2018.

Finally, we tested the moderation effect of household income and educational attainment in models 2 and 3 (Figure 5). We first include household income in model 2, indicating that people with lower household income are more likely to develop MDE than those with higher household income (β = .68, *p* < .001). This is consistent with the literature. The moderation effects on age and birth cohort effect are relatively weak (Figure 5a). Model 3 suggests that higher levels of education predict less depression this the case as Figure 5b shows that the quadric birth cohort effect is further reduced when educational attainment is taken into account. Hence, our moderation analysis supports the notion that earlier birth cohorts were more likely to be depressed as a function of their lower levels of education.

## Discussion

This paper explores the relationship between age and depression and advances the literature by investigating the temporal dimension of late-life depression, disentangling birth cohort effects from age and period effects using the hierarchical linear models (HLM), specifically HAPC models, in analyzing repeated cross-sectional national surveys. We also focused on household income and educational attainment as two major dimensions of social change, which may account for age and birth cohort effects on the prevalence of late-life depression.

Whether depressive symptoms are an inevitable concomitant of aging may be debatable. Some researchers have found that older adults are more depressed, whereas others have found the opposite, but still, others report both increasing and decreasing prevalence with age but in different age groups (Lee et al., 2021). Various hypotheses can be used to explain the divergent results. Some hypotheses view age as a life course stage sequence and predict that depression increases in old age because of the disengagement from social roles with aging (Yang, 2007). Others predict growth in depression with old age as a consequence of declines in physical health and functioning with age. Other perspectives see the relationship between old age and depression; as depending on the traits associated with survival. Specifically, differential mortality by older adults with variant traits may manifest a discrepant relationship between age and depression; for example, females live longer than males and tend to have a higher level of depression. In addition, using different measurements of depression, age compositional differences in samples, and adjustments for different sets of covariates influence findings (Schieman et al., 2001). A clinical diagnosis of major depression may tap more severe types of mental disorder, while depressive symptoms tap less serious forms of mood. Age differences in these phenomena may reflect a higher level of low-grade depressive symptoms among older adults, perhaps due to health problems and role transitions, but a lower prevalence of high-grade depressive disorders.

Another contributor to this ambiguity and disagreement is the confounding of age changes and birth cohort differences in many cross-sectional data and research designs. It is risky to infer the real-time age trajectory of individual depression levels from cross-sectional age-depression data because age and birth cohort differences are confounded. Our analysis using the HAPC model provides a more robust characterization of the relationship between age and depression while accounting for some covariates, such as income and education. We found that depression declines as older adults age after adjusting for birth cohort effects. The birth cohort-driven change in the association between age and depression manifests the cumulative exposure to individual and social risk factors. Studies consistent with our findings suggest that the divergent age-depression relationship may be related to the beneficial effects of retirement on mental health in older adults (Oksanen et al., 2011). It seems evident in our study the finding that depression decreases with age in the 70+ age groups, is likely an indicator of a “healthy survivor effect.”

Our results show that the rise in late-life depression is not purely a chronological aging-related phenomenon, but it is also strongly birth cohort driven. Extensive life course literature has given insight into the importance of individual biography intersecting with historical contexts and social changes. Birth cohorts experience constant changes in historical and social surroundings and living conditions. In this study, recent birth cohorts (those born later in the 20th century) reported higher levels of depression, and the quadratic cohort effect indicates that the positive birth cohort effect is small for the earlier birth cohorts born before 1935, with prevalence rising quickly thereafter. Various biological, psychological, and social factors contribute to late-life depression. A number of studies report that disability increases the odds of depression among the elderly (Verhaak et al., 2014). Lin et al. (2012) found that younger cohorts of older adults are becoming more disabled, net of aging and period effects, which is consistent with our study findings because of the close relationship between disability and late-life depression. In addition, Cabello et al. (2021), investigating potential age, period, and birth cohort effects on the prevalence of suicide ideation in the aging European population, concluded that more recent birth cohorts had higher levels of suicide ideation. It is well-known that suicide ideation is a significant adverse outcome of late-life depression. This birth cohort trend in suicide ideation is consistent with our study findings on late-life depression and fixed birth cohort effects.

The random birth cohort effects were dramatically lower for seniors belonging to the generation of “Parents of baby boomers” (born 1919–1940) (Statistics Canada, 2012). This generation has been referred to as the “Greatest Generation” in Canada. On the one hand, that generation was marked by great economic prosperity and significant technological advances, such as the widespread use of radio and telephone. As expected, social and technological innovations can create prosperous societies and better living conditions, which undoubtedly benefit individuals’ mental health. This could explain our findings that random cohort effects declined between the 1910s and 1920s birth cohorts. On the other hand, the random cohort effects show a steep rise in depression for people born between 1925 and 1940. The “Great Crash” in 1929 sparked a massive financial panic, and it was followed by the Great Depression of 1930 and beyond, when many companies went out of business and unemployment was rampant (Statistics Canada, 2014). People born between 1925 and 1940 experienced a period of economic chaos in childhood and adolescence. Unfavorable childhood conditions both in the family and the wider society can exert adverse effects on mental health, which may last throughout the whole life course (Xie et al., 2018). For seniors 65+ born between the 1940s and 1950s, the random cohort effects still remain at a high level. The World War II generation (born 1941–1945) suffered further increases in the level of late-life depression throughout their lives, perhaps due to restricted living conditions and the effects of conscription and lives lost on family life. Even though the war was not fought in Canada but overseas, there is no doubt children born in this period would have experienced childhood trauma as a result of World War II. Then there is the reintegration of parents returning from war. In line with our findings, Rzeszutek et al. (2020) reported that the World War II relate events have a direct positive association with the depressive symptoms among survivors of World War II. All of the above historical events constitute risk factors for late-life depression affecting specific birth cohorts.

Our findings reveal considerable irregular changes in the prevalence of depression over the past few decades, which generally indicates an absence of period effects. However, there were some period effects peaks occurring around 2001, 2008, and 2012 that may be related to more recent historical events. The tragic September 11 attacks in 2001 not only had an effect in the United States but also in Canada, with increased security concerns and heightened anti-terrorism action. Unsurprisingly, a large number of people may develop substantial psychological morbidity in the aftermath of terrorist attacks even though events were not directed directly at them (DiGrande et al., 2008). The large period effect in 2008 may be related to the global financial crisis and recession of 2008 and 2009, a period characterized by increased unemployment, falling prices, and low incomes. What is clear from previous studies is that the fall-out from volatile economic events have been found to increase the prevalence of mental disorders, including depression (Amiri, 2021; Ridley et al., 2020). Likewise, the year 2011 and 2012 also saw a roller-coaster ride for the Canadian stock market, and 2011 was a year of great political, economic, and social turmoil in much of the world. Adverse economic and political events he a clear impact on mental health is clear.

Our moderation analysis found evidence that more recent birth cohorts attained higher levels of education that reduced their rates of depression. This finding is in line with Mirowsky and Ross’s (1992) hypothesis of “age as a historical trend.” They predicted that the rise in depression in old age is due to older people being less educated. Higher levels of education are one of the major factors in the substantial historical processes that have brought more favorable social and economic conditions to recent generations (Mirowsky & Ross, 1992).

This paper shares the limitations of all studies that use APC models. Future studies can use more robust and reliable measures to assess the age, period, and birth cohort effects. In particular, period and birth cohort could be perhaps better defined in social and cultural terms rather than fixed chronological periods as we did here. In addition, more covariates can be incorporated in future research exploring the age trajectories of major depression among older adults. While repeated cross-sectional studies are better than single cross-sectional studies however high-quality longitudinal studies are better again.

Finally, an important implication of the current study is that studies evaluating the prevalence of depression in older adults should consider period and birth cohort effects in addition to chronological age or life stage. Policy interventions should aim at improving the mental health for successful aging and focusing on these three temporal dimensions of age, period, and birth cohort. It is important to recognize that early life experiences can have a significant impact on mental health in later life. To quote the proverbial expression, “The child is father to the man.”

## Supplemental Material

sj-docx-1-isp-10.1177_00207640221141785 – Supplemental material for Age, period and cohort effects in depression prevalence among Canadians 65+, 1994 to 2018: A multi-level analysis Click here for additional data file. Supplemental material, sj-docx-1-isp-10.1177_00207640221141785 for Age, period and cohort effects in depression prevalence among Canadians 65+, 1994 to 2018: A multi-level analysis by Guang Yang and Carl D’Arcy in International Journal of Social Psychiatry
