# Supplementary material for: Age, period and cohort effects in depression prevalence among Canadians 65+, 1994 to 2018: A multi-level analysis
Source: Int J Soc Psychiatry. 2022 Dec 7;69(4):885–94. doi: 10.1177/00207640221141785 (PMC10248299; doi:10.1177/00207640221141785)
Supplement: sj-docx-1-isp-10.1177_00207640221141785 – Supplemental material for Age, period and cohort effects in depression prevalence among Canadians 65+, 1994 to 2018: A multi-level analysis [file sj-docx-1-isp-10.1177_00207640221141785.docx]

Table S1. Collection of survey data on Major Depression and in the Provinces and Territories of Canada and survey instrument, Survey/year, 1998-2018

| Survey, Year | BC | AB | SK | MB | ONT | QUE | NB | NS | PEI | NLFD | YT | NT | NU* | Sample Size | Measure |
| --- | --- | --- | --- | --- | --- | --- | --- | --- | --- | --- | --- | --- | --- | --- | --- |
| NPHS, 1994/1995 | √ | √ | √ | √ | √ | √ | √ | √ | √ | √ | × | × | × | 2963 | CIDI-SFMD |
| NPHS, 1996/1997 | √ | √ | √ | √ | √ | √ | √ | √ | √ | √ | × | × | × | 12350 | CIDI-SFMD |
| NPHS, 1998/1999 | √ | √ | √ | √ | √ | √ | √ | √ | √ | √ | × | × | × | 2699 | CIDI-SFMD |
| CCHS, 2001 | √ | √ | √ | √ | √ | √ | √ | √ | √ | √ | √ | √ | √ | 23375 | CIDI-SFMD |
| CCHS, 2003 | × | √ | × | × | √ | × | √ | × | √ | √ | √ | √ | √ | 9278 | CIDI-SFMD |
| CCHS, 2005 | √ | √ | √ | × | × | √ | × | √ | √ | × | × | × | × | 13816 | CIDI-SFMD |
| CCHS, 2007/2008 | × | √ | × | × | × | √ | √ | √ | × | × | × | × | × | 9403 | CIDI-SFMD |
| CCHS, 2008/2009 | √ | √ | √ | √ | √ | √ | √ | √ | √ | √ | × | × | × | 15580 | CIDI-SFMD |
| CCHS, 2009/2010 | √ | √ | √ | × | × | √ | × | × | √ | × | × | × | × | 12946 | CIDI-SFMD |
| CCHS, 2010 | √ | √ | √ | × | × | √ | × | × | √ | × | × | × | × | 6659 | CIDI-SFMD |
| CCHS, 2011/2012 | × | × | √ | × | × | × | √ | √ | √ | √ | × | × | × | 5460 | CIDI-SFMD |
| CCHS, 2012 | × | √ | √ | × | × | × | √ | √ | √ | √ | × | × | × | 3929 | CIDI-SFMD |
| CCHS, 2013/2014 | × | × | × | √ | × | √ | × | √ | √ | √ | × | × | × | 11208 | CIDI-SFMD |
| CCHS, 2014 | × | × | × | √ | × | √ | × | √ | √ | √ | × | × | × | 5781 | CIDI-SFMD |
| CCHS, 2015/2016 | × | × | √ | √ | √ | × | √ | √ | √ | √ | × | √ | × | 14115 | PHQ-9 |
| CCHS, 2017/2018 | × | × | × | × | × | × | × | × | √ | × | √ | × | × | 684 | PHQ-9 |

Abbreviations – Province: BC = British Columbia, AB = Alberta, SK = Saskatchewan, MB = Manitoba, ONT = Ontario, Que = Quebec, NB = New Brunswick, NS = Nova Scotia, PEI = Prince Edward Island, NLFD = Newfoundland and Labrador, YT = Yukon Territory, NT = Northwest.

CIDI-SFMD = Composite Diagnostic Inventory Short Form for the major depressive episode; PHQ-9 = Patient Health Questionnaire.

Footnote* Nunavut became a separate territory of Canada in 1999.


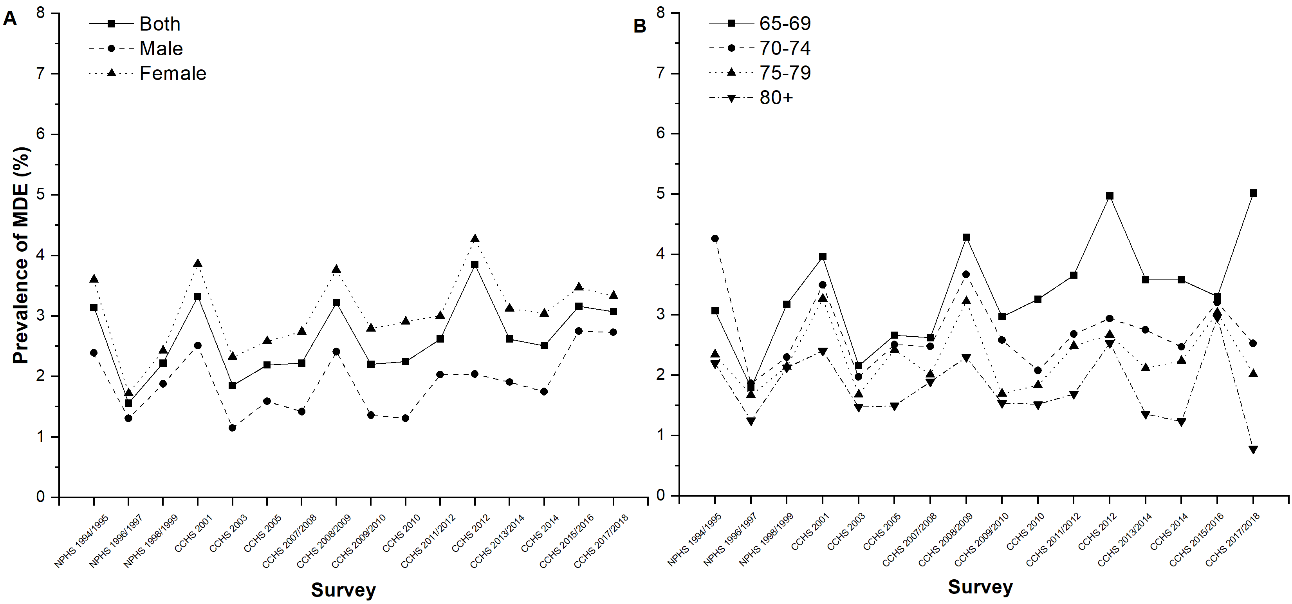


**Figure S1** **A & B.** Trends in the prevalence of MDE by gender (A) and by age group (B) in 16 waves of National Population Health Survey (NPHS) and Canadian Community Health Survey (CCHS) from 1994 to 2018 among Canadian older adults.


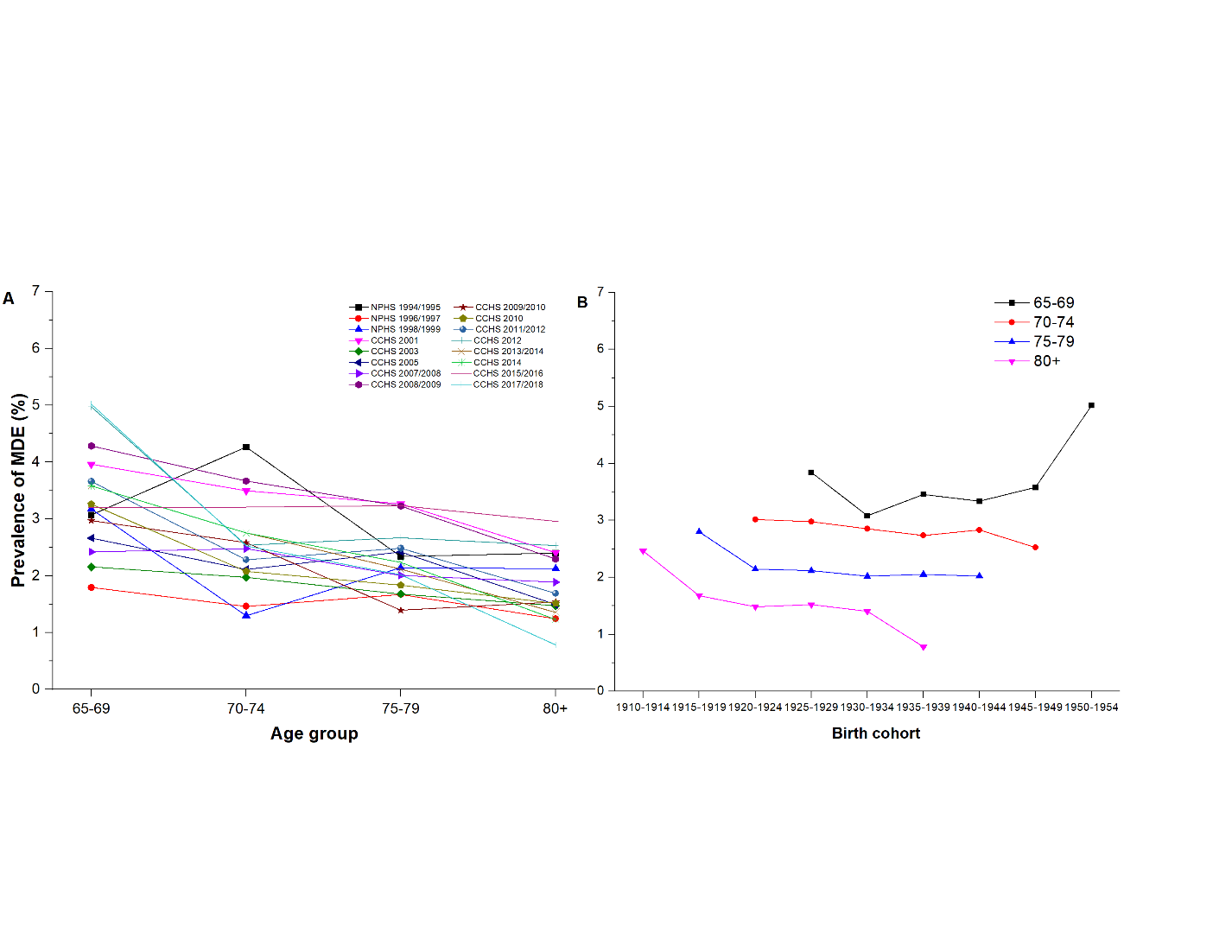


**Figure S2 A & B** Age-specific prevalence by period (A) and cohort-specific prevalence by age group (B) of MDE in Canada, 1994 to 2018.
